# Ethylenediurea (EDU) Affects the Growth of Ozone-Sensitive and Tolerant Ash (*Fraxinus excelsior*) Trees under Ambient O_3_ Conditions

**DOI:** 10.1100/tsw.2007.21

**Published:** 2007-03-21

**Authors:** Elena Paoletti, Nicla Contran, William J. Manning, Francesco Tagliaferro

**Affiliations:** ^1^ IPP-CNR, Via Madonna del Piano 10, I-50019 Sesto Fiorentino, Florence, Italy; ^2^ Department of Environmental Sciences, University of Milano-Bicocca, Piazza della Scienza 1, Milan, Italy; ^3^ Department of Plant, Soil and Insect Sciences, University of Massachusetts, Amherst, MA 01003-9320, USA; ^4^ PLA, Corso Casale 476, I-10128 Turin, Italy

**Keywords:** European ash, ethylenediurea, forest, growth, mycorrhizae, tropospheric ozone

## Abstract

Adult ash trees (*Fraxinus excelsior* L.), known to be sensitive or tolerant to ozone, determined by presence or absence of foliar symptoms in previous years, were treated with ethylenediurea (EDU) at 450 ppm by gravitational trunk infusion over the 2005 growing season (32.5 ppm h AOT40). Tree and shoot growth were recorded in May and September. Leaf area, ectomycorrhizal infection, and leaf and fine root biomass were determined in September. EDU enhanced shoot length and diameter, and the number and area of leaves, in both O_3_-sensitive and tolerant trees. However, no EDU effects were recorded at the fine root and tree level. Therefore, a potential for EDU protection against O_3_-caused growth losses of forest trees should be evaluated during longer-term experiments.

## INTRODUCTION

Much has been written about the effects of $O_3$ on growth of forest trees, but conclusive proof that ambient levels of $O_3$ affect growth of forest trees remains elusive, usually because the experimental techniques do not allow extrapolation to realistic conditions[1]. Ethylenediurea (N-[2-(2-oxo-1-imidazolidinyl)ethyl]-N'-phenylurea), abbreviated as EDU, used as a foliar spray or soil/potting medium drench, is systemic and persistent in plants, and has been used to prevent foliar $O_3$ injury and determine $O_3$ effects on growth and yield of many herbaceous plants[1,2,3] and some woody plants[4,5,6,7,8,9,10,11,12]. Gravitational trunk infusion of EDU has been demonstrated to prevent foliar $O_3$ injury on sensitive ash (*Fraxinus excelsior* L.) trees[13]. We report here additional results from the same field experiment where adult trees, considered to be either $O_3$-sensitive or -tolerant, were gravitationally infused with EDU to determine effects on tree, shoot, and fine root growth.

## MATERIALS AND METHODS

Six $O_3$-sensitive (symptomatic) and six $O_3$-tolerant (asymptomatic) adult ash trees, determined by presence or absence of foliar symptoms in previous years, were gravitationally infused with 450 ppm ethylenediurea (EDU) or water at 3-week intervals from May to September, 2005. AOT40 over the period was 32.5 ppm h. The experimental site was located at the 34-ha “Millerose” park in Turin, Italy. Details about infusion methodology, site characteristics, and EDU protection from $O_3$ visible injury are presented elsewhere[13].

Measurements of tree and shoot growth were carried out in May and September, while biomass, leaf area, and fine root mycorrhizal infection were determined only in September. Tree diameter was measured at breast height by means of a calliper. The points of measurements were labelled to reduce the error from May to September. Tree height was measured with a clinometer (Model CM360PA, Silva, Sweden). Shoot growth was measured on one 1-year-old sun shoot per tree, from the lower crown part. The shoots were the terminal ones of a lateral branch and were randomly selected. The total number of leaves and leaflets per shoot was counted. Shoot length and base diameter were recorded by means of a ruler and a digital calliper, respectively, with 0.1-mm accuracy. In September, 3 shoots per plant were collected. Total leaflet area per shoot was determined with an AM300 area meter (ADC, BioScientific Ltd, Herts UK). Shoot biomass was determined by oven-drying at 65 °C until a constant weight was reached. Leaflet mass per unit of leaflet area (LMA) and leaflet water content (LWC = (fresh weight-dry weight)/dry weight) were calculated

Fine roots (diameter<2 mm) were sampled in standard cores of soil (500 ml). Four cores per tree were collected 40 cm far from the trunk along the cardinal points and joined in a single sample. Roots were attributed to *F. excelsior* with the help of morphological and anatomical observations[14]. *F. excelsior* fine roots were analyzed with a Wild M8 (16x) stereomicroscope by means of the gridline intersect method[15] adapted to ectomycorrhizae[16]. Infected, dead and total tips were counted. Fine root length was determined using the Newman’s formula[17]. Fine root biomass, after careful rinsing with tap water for removal of adhering soil particles, was determined by oven-drying at 65 °C until a constant weight was reached.

The statistical unit was the single tree. After checking for normality, data were analyzed using a two-way (EDU *x* tree $O_3$-sensitivity) analysis of variance (Statistica 6.0, StatSoft, Tulsa, OK).

## RESULTS

EDU significantly reduced the abscission of leaves and induced a larger increment of the shoot length and diameter over the growing season, compared to the water-infused trees (Table 1). The $O_3$-sensitive trees had higher DBH increment and leaf abscission than the $O_3$-tolerant trees. EDU increased the leaf area and decreased the leaflet water content (Table 2). The latter effect was due to the LWC strong reduction in the $O_3$-sensitive trees. No EDU effect was recorded at the fine root level (Table 3). The $O_3$-sensitive trees showed lower fine root biomass and length, and thus less tips than the tolerant trees.

**TABLE 1**
**Means (±SE) and Levels of Significance of a Two-Way ANOVA for the Effects of EDU (Water vs. EDU-Infused Trees) and Tree $O_3$ Sensitivity (Symptomatic vs. Asymptomatic) on the Increments (September vs. May) in: Diameter at Breast Height (DBH), Tree Height (H), Leaf Number per Shoot (Leaf No.), Leaflet Number per Shoot (Leaflet No.), Shoot Length (SL), and Shoot Diameter (SD), in Adult *Fraxinus excelsior* Trees**

| Infusion | Tree Sensitivity | DBH Increment (%) | H Increment (%) | Leaf No. Increment (%) | Leaflet No. Increment (%) | SL Increment (%) | SD Increment (%) |
|---|---|---|---|---|---|---|---|
| EDU | Symptomatic | 8.0 ± 1.8 | 2.5 ± 2.5 | -6.2 ± 2.3 | -7.9 ± 1.7 | 17.5 ± 8.8 | 11.1 ± 2.7 |
| EDU | Asymptomatic | 2.0 ± 0.8 | 1.1 ± 1.0 | 1.8 ± 0.9 | 2.8 ± 2.5 | 17.5 ± 5.5 | 15.3 ± 0.2 |
| WATER | Symptomatic | 5.1 ± 2.7 | 0 | -7.7 ± 1.0 | -10.6 ± 1.9 | 6.7 ± 1.2 | 6.0 ± 2.0 |
| WATER | Asymptomatic | 1.0 ± 0.9 | 2.4 ± 4.0 | -2.8 ± 3.5 | -6.9 ± 2.4 | 7.7 ± 4.4 | 4.6 ± 2.5 |
| EDU | | 0.182 | 0.898 | 0.036 * | 0.100 | 0.060 + | 0.024 * |
| Tree Sensitivity | | 0.047 * | 0.577 | 0.005 ** | 0.120 | 0.194 | 0.730 |
| EDU x Tree Sensitivity | | 0.868 | 0.372 | 0.860 | 0.061 + | 0.083 | 0.506 |

***, $P \leq 0.001$; **, $P \leq 0.01$; *, $P \leq 0.05$; +, $P \leq 0.1$; NS, $P > 0.1$.

**TABLE 2**
**Means (±SE) and Levels of Significance of a Two-Way ANOVA for the Effects of EDU (Water vs. EDU-Infused Trees) and Tree $O_3$ Sensitivity (Symptomatic vs. Asymptomatic) on: Total Leaf Area and Biomass, Leaflet Mass per Unit Leaflet Area (LMA) and Leaflet Water Content (LWC) in Shoots of Adult *Fraxinus excelsior* Trees Measured at the End of the Growing Season**

| Infusion | Tree Sensitivity | Total Leaf Area $cm^2$ | Total Leaf Biomass g | LMA mg $cm^{-2}$ | LWC g $H_2O$ g leaflet $dw^{-1}$ |
|---|---|---|---|---|---|
| EDU | Symptomatic | 1368 ± 119 | 13.4 ± 3.8 | 5.6 ± 0.6 | 1.64 ± 0.12 |
| EDU | Asymptomatic | 1483 ± 85 | 17.9 ± 6.1 | 6.2 ± 0.8 | 2.35 ± 0.29 |
| WATER | Symptomatic | 951 ± 103 | 9.1 ± 2.4 | 6.1 ± 1.2 | 2.67 ± 0.29 |
| WATER | Asymptomatic | 1181 ± 305 | 15.0 ± 6.0 | 7.4 ± 0.4 | 2.28 ± 0.20 |
| EDU | | 0.053 + | 0.493 | 0.279 | 0.074 + |
| Tree Sensitivity | | 0.325 | 0.329 | 0.251 | 0.515 |
| EDU x Tree Sensitivity | | 0.739 | 0.895 | 0.650 | 0.045 + |

***, $p \leq 0.001$; **, $p \leq 0.01$; *, $p \leq 0.05$; +, $p \leq 0.1$; ns, $p > 0.1$.

**TABLE 3**
**Means (±SE) and Levels of Significance of a Two-Way ANOVA for the Effects of EDU (Water vs. EDU-Infused Trees) and Tree $O_3$ Sensitivity (Symptomatic vs. Asymptomatic) on: Biomass, Total Number of Tips, Percent of Dead Tips and Mycorrhizal Infection, and Length of Fine Roots (diameter<2 mm) in a Standard Volume (500 ml) of Soil collected under Adult *Fraxinus excelsior* Trees at the End of the Growing Season**

| Infusion | Tree Sensitivity | Biomass g | Total Tips No. | Dead Tips % | Mycorrhizal Infection % | Length cm |
|---|---|---|---|---|---|---|
| EDU | Symptomatic | 0.22 ± 0.07 | 163 ± 64 | 8.4 ± 1.2 | 83.5 ± 0.1 | 478 ± 212 |
| EDU | Asymptomatic | 1.20 ± 0.59 | 1323 ± 549 | 9.3 ± 1.1 | 81.5 ± 0.1 | 2530 ± 968 |
| WATER | Symptomatic | 0.40 ± 0.11 | 291 ± 130 | 7.5 ± 0.1 | 81.2 ± 0.1 | 839 ± 400 |
| WATER | Asymptomatic | 1.09 ± 0.37 | 727 ± 237 | 6.6 ± 0.1 | 87.0 ± 0.1 | 2122 ± 1120 |
| EDU | | 0.923 | 0.522 | 0.747 | 0.775 | 0.979 |
| Tree Sensitivity | | 0.046 * | 0.055 + | 0.691 | 0.628 | 0.097 + |
| EDU x Tree Sensitivity | | 0.692 | 0.332 | 0.869 | 0.564 | 0.672 |

***, $p \leq 0.001$; **, $p \leq 0.01$; *, $p \leq 0.05$; +, $p \leq 0.1$; ns, $p > 0.1$.

## DISCUSSION

EDU enhanced shoot length and diameter, and reduced leaf abscission. The stimulating effect of EDU on shoot growth did not translate into a stimulation of growth at the fine root and whole tree level, during one growing season. Overall, EDU effects were slight, and not enough to significantly affect the total leaf biomass. Increased growth in the EDU-treated plants over the non-EDU-treated ones has been reported in several species (*Phaseolus vulgaris*[18], *Populus nigra*[12], *Trifolium subterraneum*[19], *Pinus taeda*[8], *Vigna radiata*, [20] *Triticum aestivum*[21]).

Symptomatic trees did not show a marked reduction in growth compared to the asymptomatic trees. At the shoot level, we recorded a stimulation of leaf abscission. Part of the photosynthate may have been used to prevent or repair foliar damage rather than going toward growth[22,23], even if visible $O_3$ injury symptoms are not necessarily correlated with adverse effects on tree growth[24,25]. In the long-term, such shifts in carbon allocation may damage the below-ground system[26] and explain why we recorded the most negative effects on the $O_3$-sensitive fine roots. In contrast, tree diameter increased more in the $O_3$-sensitive trees, which may be the result of their younger age (16 vs. 31 year old in sensitive and tolerant trees, respectively), although the diameter at breast height did not differ significantly (13 ± 1 cm vs. 17 ± 5, respectively).

In conclusion, gravitational infusion of EDU resulted in stimulation of shoot growth in both $O_3$-sensitive and tolerant ash trees exposed to elevated ambient $O_3$ concentrations (32.5 ppm h AOT40). Ainsworth et al.[11] found that treatment with EDU provided protection against visible injury in poplars. As no effect on growth was found, they suggested that EDU may be incapable of protecting trees against long-term damage. Our study with *Fraxinus excelsior*, together with studies on *Gleditsia triacanthos*[9], *Prunus serotina*[7], and *Populus nigra*[12], have demonstrated that EDU can provide protection against visible injury[13], as well as against growth reductions. As no effects on fine roots and tree growth were detected in one growing season, the protection of EDU on $O_3$-impaired forest growth should be evaluated over long-term experiments.

## ACKNOWLEDGMENT

Thanks to Federica Spaziani, Mario Palenzona, Annamaria Ferrara,, Renzo Lencia, Michele Carnino, Elena Addario, and Giorgia Battaglia, who kindly provided logistic support during EDU applications and measurement campaigns; and to Regione Piemonte (project FORMEDOZONE, Interreg III B) for funding the study of ozone effects on ash.
